# Maternal effects on postembryonic neuroblast migration in *Caenorhabditis elegans*

**DOI:** 10.1093/g3journal/jkaf151

**Published:** 2025-06-30

**Authors:** Hoikiu Poon, Chaogu Zheng

**Affiliations:** School of Biological Sciences, The University of Hong Kong, Hong Kong SAR, China; School of Biological Sciences, The University of Hong Kong, Hong Kong SAR, China

**Keywords:** *C. elegans*, maternal effects, *dpy-19*, Q neuroblast, neuronal migration, maternal rescue, postembryonic development, neurodevelopment, WormBase

## Abstract

Maternal-effect genes mostly regulate early embryogenesis as their mRNAs or proteins are deposited into the oocytes to function during early embryonic development before the onset of zygotic transcription. Here, we report a case where a maternal-effect gene regulates postembryonic neuroblast migration long after the early embryonic stages. We found that the defects of the Q neuroblast migration in *Caenorhabditis elegans* mannosyltransferase *dpy-19* mutants can be rescued by a maternal copy of the gene. Maternal *dpy-19* mRNAs are deposited into the oocytes and persist throughout embryonic development into the Q cells to regulate their migration in early larval stages. These mRNAs appeared to be remarkably stable, since long-term developmental arrest, changing the 3′UTR sequence, and mutations in genes involved in RNA binding and modification all had weak effects on the maternal rescue of the neuroblast migration defects. Since the defects can also be rescued by a zygotic copy of *dpy-19(+)*, our results suggest that postembryonic neurodevelopment is redundantly regulated by maternal and zygotic copies of the same gene.

## Introduction

Maternal effect refers to a phenomenon in which the phenotype of an organism is controlled not by its own genotype but by the genotype of its mother. Maternal-effect genes are often essential for early developmental processes, since they code for RNAs and proteins important for early embryonic development before zygotic transcription takes place (Wolf and Wade 2009; Mitchell 2022). A subset of maternal-effect genes does extend their influence into postembryonic development by controlling germline development. For example, maternally deposited *oskar* mRNAs in *Drosophila* eggs (Lehmann 2016) and *bucky ball* mRNAs in zebrafish oocyte (Bontems et al. 2009) help establish the germ plasm, which is critical for germ cell specification. In *Caenorhabditis elegans*, maternal *pie-1* prevents somatic differentiation in early embryonic germline cells by repressing transcription (Seydoux et al. 1996), and the maternal *mes-2/3/6* genes regulate H3K27 methylation and are essential for germline development (Xu et al. 2001; Bender et al. 2004). Loss of these genes in the mother results in sterility in the offspring. However, very few studies reported maternal-effect genes that regulate somatic cell differentiation in postembryonic development.

One example is the *clk-1* gene in *C. elegans*, which codes for a hydroxylase involved in the biosynthesis of ubiquinone (coenzyme Q10). The *clk-1* mutants have slower developmental rate due to the lack of ubiquinone, but the homozygous mutants produced by a heterozygous mother had a normal growth rate because the persistence of maternally deposited CLK-1 proteins enabled the synthesis of sufficient amounts of ubiquinone during development (Burgess et al. 2003). In mammals, maternal nutrition during gestation affects postnatal development (Kusin et al. 1992; Hoffman et al. 2016), and placenta-specific insulin-like growth factor 2 (IGF2) expression modulates fetal growth and contributes to postnatal metabolic homeostasis (Constancia et al. 2002; Lopez-Tello et al. 2023). While these examples are mostly related to metabolism, instances of maternal-effect genes regulating neuronal differentiation in postembryonic development are rare.

**Fig. 1.**
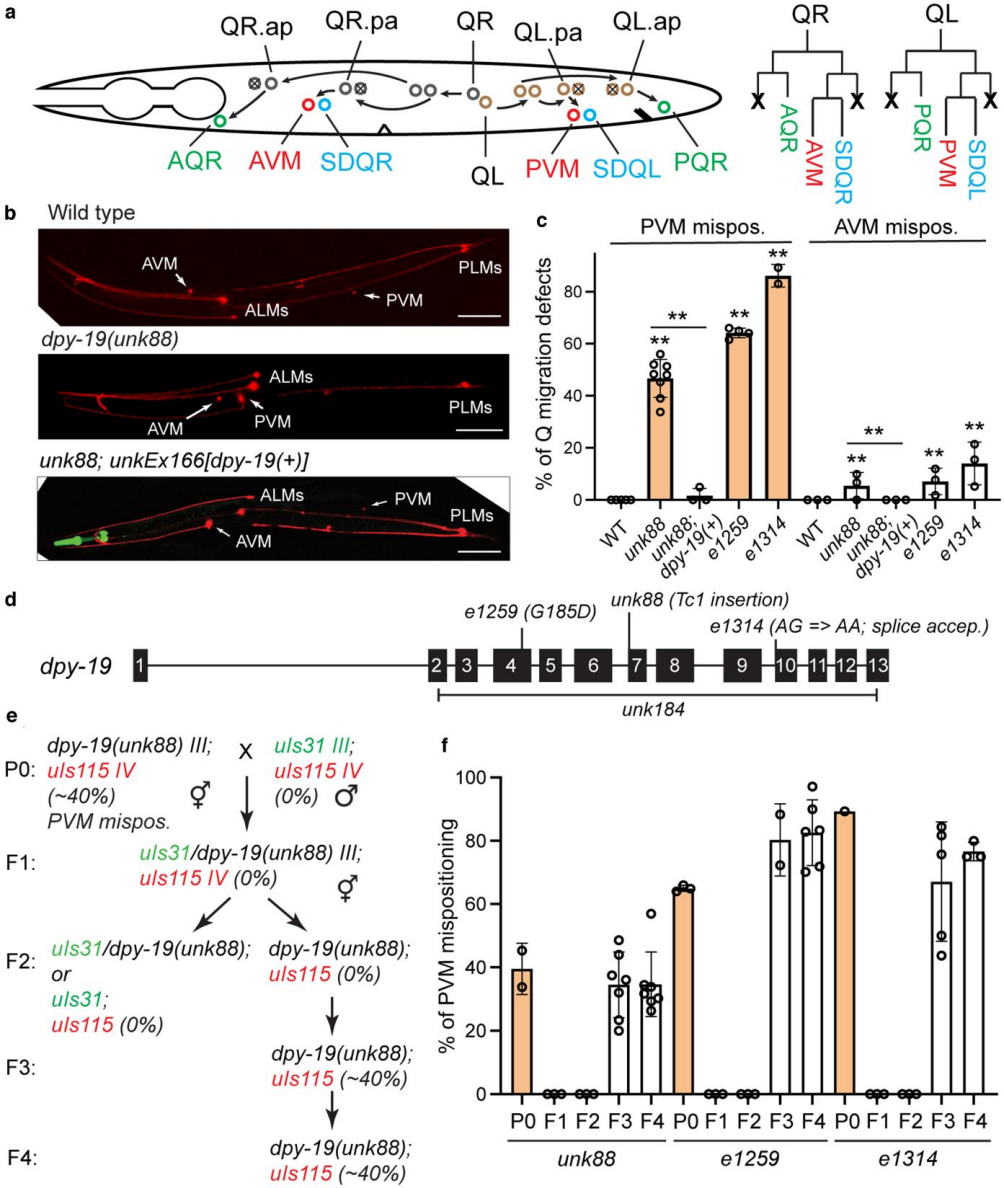
PVM positioning defects in *dpy-19* mutants are maternally rescued. a) A schematic cartoon depicting the Q lineage and the migratory route of the Q neuroblasts and their descendants. b) PVM is mispositioned to a more anterior position in *dpy-19(unk88)* mutants compared to the wild-type animals. This defect is rescued by an extrachromosomal array carrying a wild-type copy of *dpy-19*. TRNs are visualized using the reporter transgene *uIs115[mec-17p::TagRFP]*. Scale bar = 100 μm. c) Percentage of animals showing PVM and AVM mispositioning phenotype as a result of QL and QR migration defects, respectively. At least 200 animals were examined for each strain in each replicate except for the *dpy-19(+)* rescue experiment, for which >70 transformants were examined. d) Gene structure of *dpy-19* and the molecular lesion caused by various alleles. e) A cross scheme for maternal rescue experiment. *uIs31[mec-17p::GFP]* is integrated on chrIII and used as a marker for chrIII not carrying the *dpy-19* mutation. f) Percentage of animals showing PVM mispositioning in each generation according to the labeling in e) for various *dpy-19* alleles. For *unk88* and *e1259*, *N* > 100; for *e1314*, *N* > 65.

In this study, we report one such case where a maternal-effect gene regulates postembryonic neuroblast migration in *C. elegans*. Previous studies of Q lineage development established a model for neuroblast migration in *C. elegans*, as the Q neuroblasts migrate, divide, and give rise to 3 types of neurons, including the mechanosensory touch receptor neurons (TRNs), oxygen-sensing neurons, and 2 interneurons (Middelkoop and Korswagen 2014) (Fig. 1a). *dpy-19*, which codes for a mannosyltransferase, is required for the initial polarization of the Q cells by mediating the C-mannosylation of MIG-21 (a small transmembrane protein with thrombospondin type 1 repeats [TSR]), and the glycosylation is essential for the secretion of soluble MIG-21 (Honigberg and Kenyon 2000; Buettner et al. 2013). DPY-19 also catalyzes the mannosylation of UNC-5 (a netrin receptor), although *unc-5(−)* mutants do not show the Q cell migration defects seen in *dpy-19(−)* mutants (Honigberg and Kenyon 2000; Buettner et al. 2013). The loss of *dpy-19* led to the mispositioning of the neurons derived from the Q lineage, including the TRN subtype PVM neuron. We found that the *dpy-19* homozygous mutants derived from the heterozygous mothers showed normal positioning of Q lineage descendants, indicating normal Q cell migration. The *dpy-19* mRNAs but not proteins were maternally deposited into the oocytes and were passed down to the Q cells during embryonic development. Moreover, these maternal *dpy-19* mRNAs appeared to be remarkably stable, since long-term developmental arrest, swapping the 3′UTR with non-maternal-effect genes, and mutations in RNA stabilizing genes had only minor effects on the maternal rescue of the neuroblast migration defects. Overall, our study demonstrates that postembryonic neuronal development is safeguarded by the persistence of maternally deposited mRNAs.

## Materials and methods

### Strains and transgenes

*C. elegans* wild-type (N2) and mutant strains were maintained as previously described (Brenner 1974). Most of the experiments were performed at 20 °C on NGM plates seeded with *Escherichia coli* (OP50) as food source unless otherwise indicated. Transgene *uIs115[mec-17p::TagRFP]* was used to visualize the TRNs, and the transgene *uIs130[lad-2p::GFP]* was used to visualize SDQ neurons. *uIs31[mec-17p::GFP]*, which is integrated on chromosome III and labels the TRNs in green, was used as a chr III marker. *dpy-19(e1259)*, *dpy-19(e1314)*, *mig-21(u787)*, *cdh-4(hd40)*, *ptp-3(mu256)*, *unc-40(n324)*, *pab-2(ok1851)*, and other mutants were obtained from the Caenorhabditis Genetics Center. A list of strains used in this study can be found in Supplementary Table 1.

### Genomic mapping of *unk88*

We first used a set of chromosome markers to test their linkage with *unk88* and found that the allele was located on chr III. We then used a previously published single-nucleotide polymorphism (SNP) 2-point mapping method (Davis et al. 2005) to narrow down the genomic location of *unk88* by crossing the mutant strain with the genetically divergent CB4856 strain. This approach allowed us to map *unk88* between −19 and 4 on chr III. We then outcrossed the strain against the wild-type animals carrying *uIs115* and conducted whole-genome resequencing of the outcrossed strain and identified a 4-nucleotide (nt) insertion (III: 8661495 GTA => GTACATA) in *dpy-19*. Genotyping of the *dpy-19* locus revealed the Tc1 transposon insertion, and the 4-nt insertion appeared to have resulted from the self-excision of the transposon.

To rescue the *dpy-19(unk88)* mutant phenotype, we cloned an 11.7-kb genomic fragment including a 3-kb *dpy-19* promoter and the entire coding region and a 1-kb sequence downstream of the stop codon into pUC57. We then injected the *dpy-19(+)* construct into *dpy-19(unk88); uIs115* to obtain the transgenic animals with extrachromosomal array and examined the PVM positions.

### CRISPR/Cas9-mediated gene editing

To create the *dpy-19* deletion allele, we used CRISPR/Cas9-mediated gene editing to make cuts at 2 separate sites (exons 2 and 13) of the endogenous *dpy-19* locus. The 2 target sequences were 5′-GGTAGTTGATGTATCCAACG-3′ and 5′- ATGTGTTTATCGTGGAATGT-3′, respectively. The single guide RNAs (sgRNAs) were synthesized using the NEB EnGen sgRNA Synthesis Kit (E3322V) and were injected together with recombinant Cas9 (EnGen S. pyogenes Cas9 NLS from NEB, M0646T) into the *C. elegans*. We used pCFJ104 (*myo-3p::mCherry*) as the coinjection marker, and the transformants with red muscles were genotyped for any deletion in *dpy-19*. The allele that deleted the sequence between the 2 cut sites was named *unk184*.

To create the *dpy-19* (*mig-21 3′UTR*) allele, we chose the CRISPR/Cas9 target sequence 5′-AAATAGTTTATAATGCTAAT-3′ close to the stop codon of *dpy-19* and created sgRNA targeting the site and coinjected repair template that replaced the *dpy-19* 3′UTR (5′-tttttttctattttgtttttaaatttatttatttagttccagtatttttctgtaattccaaaacgatgaaatcaaatgaaccggtactgtatgttg-3′) with the *mig-21* 3′UTR (5′-atttcggatgctttcgagaacagtctctgtctgcccatttctcacgccacgataataaaagttatcattgatc-3′). The gene editing experiment was performed by SunyBiotech (Fuzhou, China), and the resulted allele was named *syb8232*. We verified the sequence through genotyping after receiving the strain.

### RT-PCR

Young embryos of N2, *unk88*, and *e1314* were collected in multiple batches. Animals were bleached to synchronize growth and grown on OP50-seeded 100-mm NGM plates under 15 °C to ensure abundant eggs were produced. When eggs were visible inside the uterus and ideally before gastrulation (and there were little to no eggs on the plate), worms were washed and bleached to collect the eggs. RNA extraction was performed using TRIzol reagent (Thermo Fisher Scientific) according to the standard protocol. cDNAs from different samples were normalized using their *tba-1* expression level visualized on a gel. PCR against *dpy-19* was conducted using primers that bind to the exon 1 to 2 and exon 12 to 13 junctions. The PCR products were visualized on an agarose gel after electrophoresis and semiquantitatively measured by comparison between the *dpy-19* mutants and the wild type. Primer information can be found in Supplementary Table 1. Similar RT-PCR experiments were also conducted on mixed stage samples.

### smFISH

To visualize the endogenous *dpy-19* mRNAs, we designed the single-molecule fluorescent in situ hybridization (smFISH) probes against the *dpy-19* transcript using the Stellaris RNA FISH Probe Designer from Biosearch Technologies (Petaluma, California, United States) and synthesized the probes with Quasar 670 dye. The staining was conducted according to the procedures detailed in “Stellaris RNA FISH Protocol for *C. elegans*” by Biosearch Technologies. Eggs were obtained from gravid adults of N2, *unk88* and *unk184* strains. Samples were fixed in 37% formaldehyde, stored in 70% ethanol overnight, hybridized with a probe-containing hybridization buffer overnight, and then washed and imaged using the Leica DMi8 Inverted Microscope. One-cell, 2-cell, and 4-cell stage eggs were visualized and quantified for the number of RNA signals detected. Each egg was imaged using the Z-stack function and 1 image was captured every 1.5 µm. A total of 10 to 15 images were taken for each sample. As each fluorescent punctum represents individual RNA molecules, we counted all puncta from the stack of images to obtain the total number of *dpy-19* transcripts in each sample.

To observe the presence of maternal *dpy-19* mRNAs in the Q lineage, we first conducted smFISH against *dpy-19* in a strain carrying a Q cell marker *ayIs9[egl-17p::GFP]* and the colocalization of the smFISH signal with GFP served as a positive control to show that *dpy-19* is indeed expressed in the Q cell. We then grew many *dpy-19(unk184)/uIs31 III* heterozygous animals to obtain F2 animals, which could be either nonfluorescent *dpy-19(unk184)* or fluorescent *dpy-19(unk184)/uIs31* and *uIs31*. We then bleached the F2 animals to get F3 eggs, which were grown to L1 and subjected to smFISH staining for *dpy-19*. The F3 animals that are derived from the nonfluorescent F2 *dpy-19(unk184)* animals did not inherit maternal *dpy-19* mRNA (i.e. M−), and the ones from F2 *dpy-19(unk184)/uIs31* animals inherited maternal dpy-19 mRNA (i.e. M+). In fact, in the nonfluorescent F3 animals, any *dpy-19* transcripts in the Q cells should be derived from maternally deposited *dpy-19* mRNAs.

### Tests of maternal rescue under various conditions

A typical maternal cross was carried out between *dpy-19* mutant hermaphrodites and wild-type males carrying the *uIs31[mec-17p::GFP]* transgene, which is located on the same chromosome as *dpy-19*. The green F1 heterozygous *dpy-19/uIs31* mutants were allowed to self-fertilize and produce nongreen F2 *dpy-19* homozygotes, which were analyzed for PVM positioning. Both P0 parents carried *uIs115[mec-17p::TagRFP]* for the visualization of TRNs. Some F2 *dpy-19* homozygotes were picked onto fresh plates to produce F3 homozygotes, which were also examined. In the starvation experiment (Burgess et al. 2003), we bleached the F1 animals to obtain F2 eggs, which were hatched in M9 and arrested at L1 stage on NGM plates, containing no food but ampicillin (100 µg/mL), kanamycin (50 µg/mL), and streptomycin (100 µg/mL) to prevent contamination for 3, 6, 9, 12, 15, 18, 30, and 32 d at 15 °C. On the day of experiment, we flooded the plates with OP50-cotaining M9 to form a thin layer of bacteria, on which surviving animals were fed until they reached day 1 adults. We examined the F2 *dpy-19* homozygous animals without green fluorescence for PVM positioning defects.

To test the effects of *dpy-19* 3′UTR, we first crossed *dpy-19(mig-21 3′UTR)* with *dpy-19(unk184)* to obtain the heterozygotes and then examined the *dpy-19(unk184)* homozygotes (by genotyping or observing dumpy animals in the F3) for potential PVM mispositioning phenotypes. To detect the effects of mutating genes that may regulate mRNA stability, we first crossed *pab-2(ok1851)* and *gtbp-1(ax2029)* with *dpy-19(unk184)* to create the double mutants. Since *cey-4* and *imph-1* are located on the same chromosome as *dpy-19*, we generated double mutants by deleting *dpy-19* in *cey-4(ok858)* and *imph-1(tm1623)* mutants using the same gene editing method described above. We then crossed the double mutants with the corresponding single mutants (i.e. *pab-2*, *gtbp-1*, *cey-4*, and *imph-1*, respectively) and analyzed the F2 progeny for potential disruption of maternal rescue.

### Quantification of PVM mispositioning

The AQR, AVM, and SDQR neurons in the wild-type animals reside in the anterior half of the worm's body, while PQR, PVM, and SDQL reside in the posterior half. “Anterior” is defined as any point anterior to the vulva, while “posterior” is defined as posterior to the vulva. The positioning of these neurons is the result of anteriorly directed migration of QR cell and the posteriorly directed migration of QL cell. Since the effects of *dpy-19* mutations on the initial polarization of Q cells were previously characterized (Honigberg and Kenyon 2000), we used the final position of these neurons to indicate Q cell migration defects. In this study, “mispositioning” of a neuron means that an anterior neuron was observed in the posterior half of the body, or a posterior neuron was observed in the anterior half. We counted the number of animals showing mispositioned neurons in a population to obtain the penetrance of a mutant phenotype. Microscopic imaging was done on a Leica DMi8 inverted microscope equipped with a Leica K5 monochrome camera, and images were analyzed using the Leica Application Suite X software. In general, QL migration was more severely affected than that of QR in *dpy-19* mutant animals. So, we focused on the mispositioning of PVM, PQR, and SDQL neurons. To compare the penetrance across strains, we calculated the average percentage of animals showing the PVM mispositioning phenotype across at least 3 biological replicates and used a 1-way ANOVA followed by either Dunnett's tests or Tukey's honestly significant difference tests to find out statistical significance in multiple comparison.

## Results

### A transposon insertion in *dpy-19* led to Q cell migration defects

In the process of studying the genetic control of TRN development, we analyzed the *ham-2(n1332)* allele, which was previously reported to affect HSN development (Desai et al. 1988). We crossed the MT3149 *ham-2(n1332)* strain with the TRN marker *uIs115[mec-17p::TagRFP]* and segregated an autosomal allele that caused the mispositioning of the PVM neuron, which is a postembryonic TRN subtype derived from the QL cell lineage (Fig. 1a). In the wild-type animals, the PVM cell body was in the posterior half of the body, whereas in the mutants, ∼40% of the PVM was displaced anteriorly to a position close to the AVM cell body (Fig. 1b and c). This result was unexpected because *ham-2* is an X-linked gene, but the PVM phenotype was not X-linked. Using a combination of SNP mapping (Davis et al. 2005) and whole-genome resequencing, we mapped the mutation that caused PVM mispositioning to *dpy-19* and found a Tc1 insertion at the beginning of exon 7 (Fig. 1d;  Supplementary Fig. 1). The Tc1 transposon was found to be excised at a low frequency, which generates small indels (typically a 4-nt insertion) in exon 7 (Supplementary Fig. 1a). Interestingly, Tc1 was removed from the pre-mRNA likely due to the use of a cryptic splice site, which caused a frameshift deletion of 49 bp in exon 7 (Supplementary Fig. 1b). We named the *dpy-19* allele *unk88*.

Tracing back to its origin, we found that the *ham-2(n1332)* allele was reported as a transposon insertion allele generated in the *mut-2* background (Desai et al. 1988). One of the popular *mut-2* strains used in mutagenesis studies was MT3126 *mut-2(r459); dpy-19(n1347)* (Rushforth et al. 1993). We genotyped *dpy-19* in MT3126 and found a Tc1 insertion at the same site as *unk88*, suggesting that *unk88* was likely to be *n1347*. Nevertheless, to avoid confusion, we still use *unk88* to refer to the *dpy-19* allele isolated from MT3149 *ham-2(n1332)*.

Previous results found that both QL and QR lacked clear polarization in *dpy-19* mutants (Honigberg and Kenyon 2000), suggesting that the positioning of the descendants of both QL and QR lineages may be similarly affected. We found that *dpy-19(unk88)* mutants mostly caused anterior displacement of the PVM cell body and only led to the posterior displacement of AVM in a small percentage of animals, suggesting that the QL migration may be more strongly disrupted by mutations in *dpy-19* than QR (Fig. 1b and c). Since the function of *dpy-19* in Q cell polarization has been characterized before (Honigberg and Kenyon 2000), we used the final position of Q descendants (e.g. PVM) as a measurement for Q migration defects. To confirm that *dpy-19(unk88)* was indeed the phenotype-causing mutation, we injected a DNA construct containing the genomic fragment of *dpy-19(+)* into the mutants and found that the PVM mispositioning phenotype was fully rescued in the transformants (Fig. 1b and c).

### Maternal rescue of the Q migration defects in *dpy-19* mutants

When analyzing the *unk88* allele, we noticed a maternal rescue of the PVM phenotype in the *dpy-19* mutants, where homozygous *dpy-19(unk88)* animals (F2) produced by the *dpy-19(unk88)/+* heterozygous mothers (F1) did not show the PVM mispositioning. We only started to observe the PVM phenotype in the homozygous F3 generation (Fig. 1e). Interestingly, the *dpy-19(unk88)/+* heterozygotes produced by crossing homozygous hermaphrodites (P0) and wild-type males did not show the mispositioning of PVM cell body, suggesting that the phenotype was also rescued by the zygotic copy of *dpy-19(+)*. Moreover, *dpy-19(unk88)* animals produced by crossing *dpy-19* homozygous hermaphrodites with *dpy-19/+* heterozygous males showed the PVM phenotype, indicating the lack of paternal rescue (Supplementary Fig. 2a). Since the PVM mispositioning phenotype had incomplete penetrance (∼40%) in *dpy-19* mutants, we also assessed whether the maternal phenotype of homozygous mothers (F3 generation) had an influence on the penetrance of the offspring and found no clear difference between the progeny of *dpy-19* mothers with and without the PVM phenotype (Supplementary Fig. 2b and c).

Next, we compared *unk88* with 2 reference *dpy-19* alleles, *e1259* and *e1314*, for their phenotypes on Q neuroblast migration. *e1259*(G185D) is a missense allele in exon 4, and *e1314* is a splice acceptor mutation that affects the splicing of exon 10 (Fig. 1d). Both *e1259* and *e1314* alleles showed stronger defects in QL migration than *unk88*, indicated by the anterior displacement of the PVM cell body in 60% to 90% of the animals (Fig. 1c). We observed the maternal rescue of the PVM positioning defects in *e1259* and *e1314* mutants, since F2 homozygotes from the F1 *dpy-19/+* heterozygotes did not show any PVM phenotype (Fig. 1f). Moreover, ∼5% of AVM cell bodies in *unk88* mutants and 10% to 20% of AVM in *e1259* and *e1314* mutants were mispositioned to the posterior half of the animal, suggesting that QR migration was also affected, although to a lesser extent. The AVM mispositioning defects in *dpy-19* mutants were also maternally rescued (Supplementary Fig. 3).

*dpy-19* was originally identified by Brenner (1974) as a gene that, when mutated, causes the animals to be short and dumpy. Indeed, *e1259* and *e1314* mutants resulted in a temperature-dependent dumpy phenotype; when animals were grown at 20 °C or above, their lengths were significantly smaller than the wild type (Supplementary Fig. 4a and b). Importantly, the dumpy phenotype of *dpy-19* mutants was also maternally rescued (Supplementary Fig. 4c). Nevertheless, we did not observe an obvious dumpy phenotype in *unk88* mutants, suggesting that *unk88* may be a weaker allele than *e1258* and *e1314*. RT-PCR results showed that the *dpy-19* transcript levels were comparable among the wild type, the *unk88* mutants, and the *e1259* missense mutants but were reduced in the *e1314* splice acceptor mutants (Supplementary Fig. 4d).

**Fig. 2.**
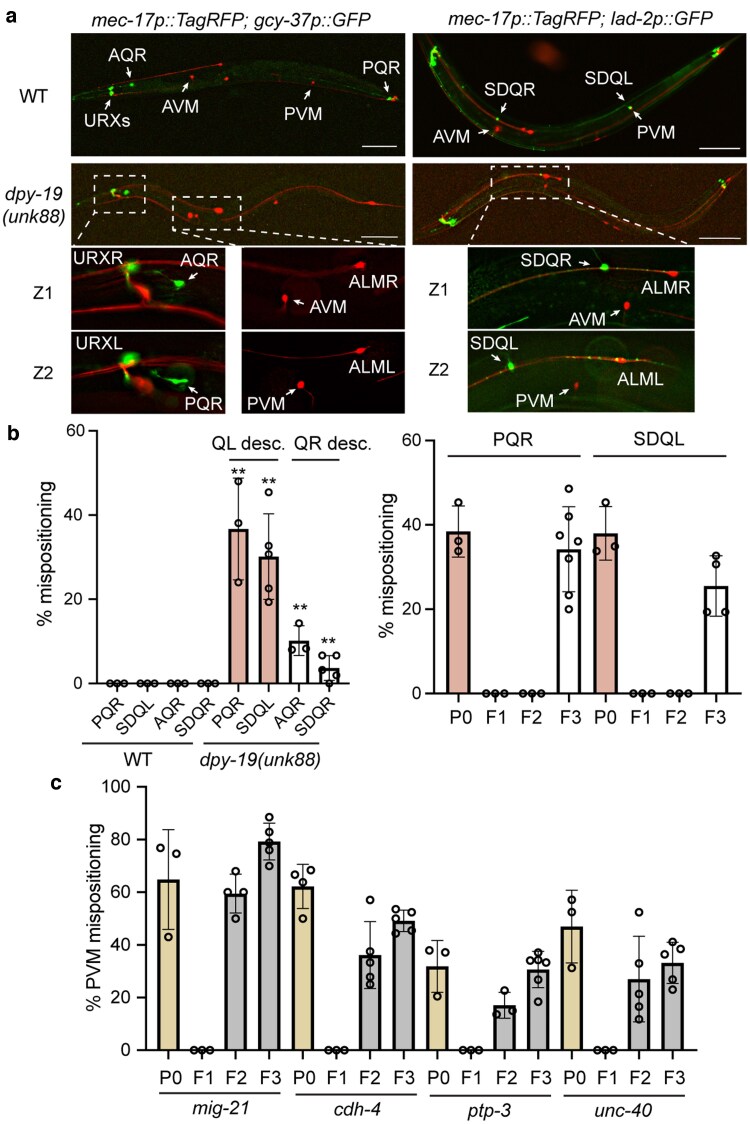
Maternal effects of dpy-19 affect the positioning of all Q lineage descendants. a) The positions of QL descendants (PVM, PQR, and SDQL) were all affected by *dpy-19(unk88)* mutation in the same animal. PQR, labeled by *iaIs25[gcy-37p::GFP]*, was normally positioned around the tail and was mispositioned to the head in *dpy-19* mutants; SDQL, labeled by *uIs130[lad-2p::GFP]*, was normally positioned in the posterior half of the animal and was mispositioned to the anterior in *dpy-19* mutants. Scale bars = 100 μm. Z1 and Z2 showed 2 focal planes of the same animal. b) Left panel shows the percentages of *dpy-19(unk88)* animals showing PQR, SDQL, AQR, and SDQR cell body mispositioning phenotypes. Right panel shows the percentages of animals showing PQR and SDQL mispositioning in each generation in a cross similar to the one in Fig. 1e. P0 and F3 animals were *dpy-19(unk88; M-)*, whereas F2 animals were *dpy-19(unk88; M+)*. Over 100 animals were examined for each cell type at each generation. c) Results of the tests for maternal effects of 4 other genes involved in QL migration using a cross similar to the one in Fig. 1e. *mig-21(u787)*, *cdh-4(hd40)*, *ptp-3(mu256)*, and *unc-40(n324)* alleles were used. Over 100 animals were examined for each strain at each generation.

In addition to AVM and PVM, the Q lineages also give rise to 4 other neurons: the oxygen-sensing neuron AQR and PQR and the interneurons SDQL and SDQR. Defects in Q cell migration would also lead to the mispositioning of these neurons. Indeed, we observed the anterior displacement of PQR and SDQL cell bodies in ∼40% of the *dpy-19(unk88)* mutants and the posterior displacement of AQR and SDQR in 5% to 10% of the mutants (Fig. 2a and b). As expected, the QL descendants (PQR, SDQL, and PVM) were often displaced together in the same animal, and the QR descendants (AQR, SDQR, and AVM) were also displaced together, confirming that mutations in *dpy-19* affected the initial migration of the Q neuroblasts. We also confirmed the maternal rescue of *dpy-19* mutants on the PQR and SDQL mispositioning phenotype, suggesting that the postembryonic development of the entire Q lineage is regulated by maternal DPY-19 (Fig. 2b).

The C-mannosyltransferase DPY-19 promotes Q cell polarization by promoting the mannosylation and solubilization of the TSR-containing membrane protein MIG-21 (Buettner et al. 2013). Mutations in *mig-21* caused a similar PVM displacement as *dpy-19* mutants (Middelkoop et al. 2012), but the PVM phenotype in *mig-21* mutants was not maternally rescued (Fig. 2c). Moreover, UNC-40/DCC receptor, PTP-3/LAR-like receptor tyrosine phosphatase, and CDH-4/Cadherin function in the same pathway as MIG-21 in regulating Q polarization (Sundararajan and Lundquist 2012; Sundararajan et al. 2014). Although their mutants all showed PVM mispositioning, none of them had maternal rescues (Fig. 2c). So far, to our knowledge, *dpy-19* appears to be the only Q cell-regulating gene that displays maternal effects.

### *dpy-19* mRNAs are maternally deposited into embryos

**Fig. 3.**
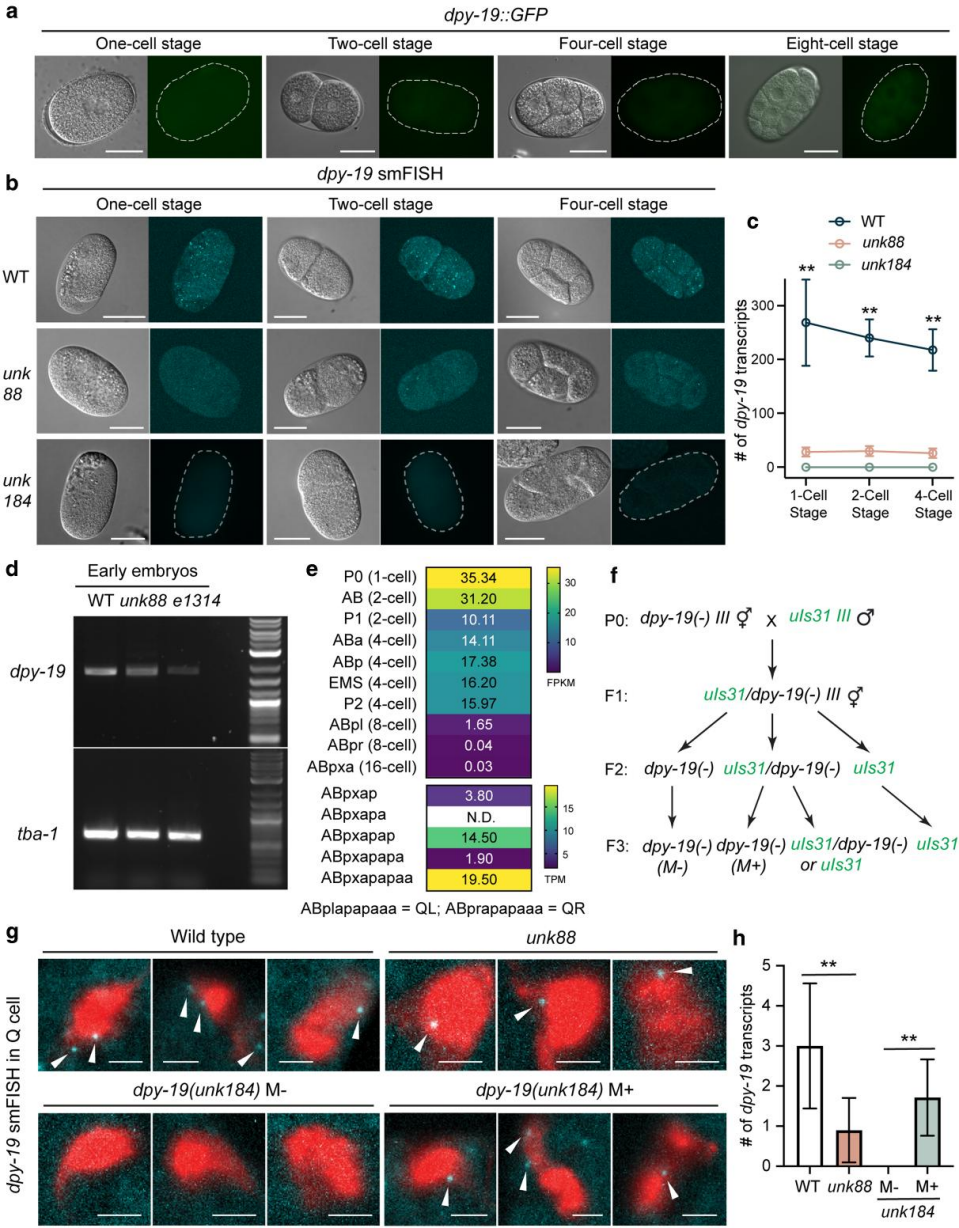
Maternally deposited *dpy-19* mRNAs persisted throughout development. a) DPY-19::GFP expressed from an endogenous GFP knock-in allele *hu257[dpy-19::gfp::SEC::3xflag]* did not show any GFP signal in early embryos. b) smFISH staining against *dpy-19* in wild-type, *unk88*, and *unk184* (the *dpy-19* deletion allele; Fig. 1d) animals at early embryonic stages before the onset of zygotic transcription. Scale bars = 20 μm. c) The number of all smFISH signals (i.e. fluorescence puncta) in each embryo counted through a Z stack of 10 to 15 pictures. Double asterisks indicate significant difference (*P* < 0.01) between the wild-type animals and each of the 2 mutants in a post-ANOVA Dunnett's test. *N* > 15 for each sample at each stage. d) RT-PCR of *dpy-19* from the cDNA library of early embryos (unlaid eggs at 32-cell stage or earlier). e) *dpy-19* expression levels extracted from published single-cell transcriptomic data of the embryos (Tintori et al. 2016; Packer et al. 2019). ABpl and ABpr lineages that give rise to QL and QR, respectively, are grouped into the same embryonic cell types as ABpx. f) The cross scheme to examine the maternal *dpy-19* mRNAs using smFISH; *dpy-19(unk184)* was used as the *dpy-19(−)* knockout allele. We included the transgene *ayIs9[egl-17p::GFP]*, a Q cell marker, in the background to facilitate the identification of the Q cells. g) smFISH against *dpy-19* in the Q cells of *dpy-19(−)* animals with or without maternal rescue according to the cross in f). The wild-type and *unk88* animals served as controls; GFP signal from *ayIs9* was pseudo-colored in red; smFISH signals were indicated by arrow heads. Scale bar = 2 μm. h) Quantification of the smFISH results from g). *N* = 15 for WT, 30 for *unk88*, 20 for M−, and 8 for M+. Double asterisks indicate statistical significance (*P* < 0.01) in a post-ANOVA Tukey's test.

Maternal effects are generally mediated by maternal deposits of proteins or mRNAs or epigenetic modifications, such as DNA methylation and histone modifications (Mitchell 2022). To explore these mechanisms, we first examined a strain with GFP knock-in at the endogenous *dpy-19* locus (Ebbing et al. 2019). The DPY-19::GFP fusion protein was reported to be functional (Ebbing et al. 2019), and we did not observe any dumpy phenotype or PVM mispositioning in the strain. Although the DPY-19::GFP signal can be observed at mid- and late-stage embryos and early larvae (Supplementary Fig. 5), we found no DPY-19::GFP signal in the early embryos, suggesting that there is no maternal deposition of DPY-19 proteins (Fig. 3a).

To check for maternal deposition of *dpy-19* mRNAs, we performed smFISH to quantitatively detect *dpy-19* mRNAs in early embryos before zygotic transcription occurs. For a negative control, we generated a *dpy-19* knockout mutant (*unk184*) through CRISPR/Cas9-mediated gene editing (Fig. 1d). The *unk184* allele was used as *dpy-19(−)* in the following studies. Using smFISH, we could clearly detect >200 *dpy-19* mRNAs in early embryos (i.e. zygote, 2-cell stage, and 4-cell stage embryos) in the wild-type animals, much reduced mRNAs levels (∼20 per embryo) in *unk88* animals, and virtually no mRNA signal in the *dpy-19* knockout mutants (Fig. 3b and c). We also conducted RT-PCR on the early embryos of up to the 8-cell stage and found *dpy-19* expression in the wild-type embryos; *unk88* and *e1314* mutants showed reduced embryonic mRNA levels (Fig. 3d). The above results indicate that *dpy-19* mRNAs are maternally deposited into the oocyte to mediate maternal effects.

Single-cell transcriptomic studies (Tintori et al. 2016; Packer et al. 2019) also confirmed *dpy-19* expression in all early embryonic cells from zygote to 4-cell stage, as well as most of the lineage precursors that give rise to the Q neuroblasts (Fig. 3e). These results at the single-cell level mapped out the lineage routes through which maternally deposited *dpy-19* mRNA could be passed to the Q neuroblasts of the offspring to produce DPY-19 proteins. To confirm this inheritance, we used smFISH to detect maternally deposited *dpy-19* mRNAs in the Q cells of *dpy-19(−)* animals produced by the *dpy-19(−)/+* heterozygous mothers and, indeed, found mRNA signals that overlapped with the Q cells (QL and QR), labeled by *ayIs9[egl-17p::GFP]* (Fig. 3f to h). These results suggested that the absence of Q cell migration defects in the F2 *dpy-19(−)* homozygous animals was due to the deposition of wild-type *dpy-19* mRNA from the heterozygous mother.

In addition to maternal deposit, *dpy-19* is also likely to be transcribed zygotically, because the mRNA level of *dpy-19* is higher in mid- and late-stage embryos compared to the 1-cell embryo according to the modENCODE data from WormBase (WS287) (Sternberg et al. 2024). By studying embryonic transcriptomes, Quarato et al. (2021) classified *dpy-19* as a “maternal stable” and “maternal and zygotic” gene, whose mRNAs level is detected in 1-cell embryos, is stably maintained in early embryos, and increases in late embryos. Zygotic expression of *dpy-19* is consistent with the zygotic rescue of PVM mispositioning phenotype in the cross progeny of *dpy-19* homozygous mothers and wild-type fathers (Fig. 1e). As controls, the classical maternal gene *mom-2*, which only functions in early embryogenesis, was classified as a “maternal cleared” gene by Quarato et al. (2021), meaning that its mRNAs were found in 1-cell embryos but were degraded in early embryos. As expected, *mig-21* mRNAs were not found in 1-cell embryos, which explained the lack of maternal effects on Q cell migration. However, *unc-40*, *ptp-3*, and *cdh-4* mRNAs were unexpectedly found in the 1-cell embryos by Quarato et al. (2021) and in the zygotes and early blastomeres by single-cell transcriptomics (Tintori et al. 2016), despite the lack of maternal effects. We suspect that these maternally deposited mRNAs may not be passed into the Q lineage precursors and contribute to Q migration in the progeny.

Lastly, to explore whether DPY-19 may act through epigenetic modifications, we searched for potential DPY-19 substrates by scanning the *C. elegans* proteomes for the “WXXWXXW” motif, which is the glycosylation target recognized by DPY-19 (Buettner et al. 2013). The search yielded 41 possible DPY-19 targets, including MIG-21 and UNC-5, but none of them had direct connections with DNA or histone modification (Supplementary Table 2). Thus, we reason that DPY-19 is unlikely to produce maternal influence via epigenetic modifications.

### Maternal *dpy-19* mRNAs are likely stabilized via multiple mechanisms throughout development

**Fig. 4.**
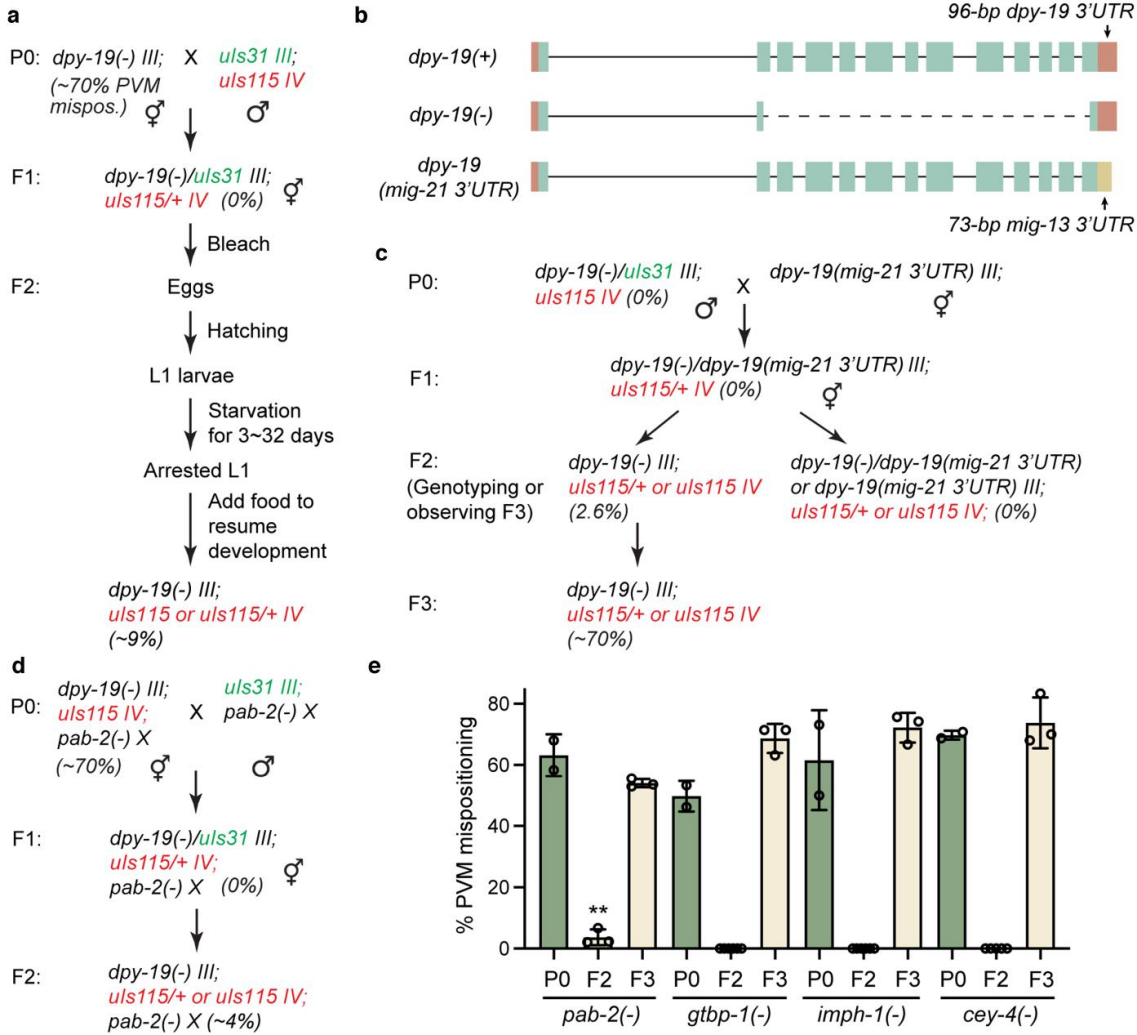
Maternal effect of *dpy-19* is robust against various perturbations. a) An experimental scheme to test the effect of long-term developmental arrest on the maternal rescue of *dpy-19(unk184)* mutants. b) Gene structure of *dpy-19(unk184)*, referred to as *dpy-19(−)*, and *dpy-19(syb8232)*, referred to as *dpy-19(mig-21 3′UTR)*, in which the *dpy-19* 3′UTR is replaced by *mig-21* 3′UTR at the endogenous locus. c) A cross scheme to test the effects of the 3′UTR swap on the maternal rescue of the PVM mispositioning defects in *dpy-19(unk184)* mutants. d) A representative scheme to test various genetic backgrounds for their impact on the *dpy-19* maternal rescue using *pab-2* as an example. e) The percentage of animals with PVM mispositioning defects in different generations (P0 and F3 represented M−, while F2 represented M+) under the *pab-2(ok1851)*, *gtbp-1(ax2029)*, *imph-1(tm1623)*, and *cey-4(ok858)* backgrounds using the cross schemes detailed in d). At least 50 animals were examined for each strain.

The passage of maternally deposited *dpy-19* mRNA from oocyte to the Q cells suggests unusual stability of the mRNAs over a period of at least 14 h (the time from fertilization to hatching, given that Q cells polarize and start to migrate shortly after hatching). We next attempted to identify the possible mechanisms that help stabilize *dpy-19* mRNA throughout embryogenesis. Inspired by a previous study (Burgess et al. 2003), we first tested whether long-term developmental arrest at the L1 stage could destabilize maternal *dpy-19* mRNA and thus disallow the maternal rescue in *dpy-19(−)* mutants. Surprisingly, arresting the *dpy-19(−)* mutants derived from heterozygous mother at L1 stage for ≤30 d before recovery did not affect the maternal rescue at all, and we observed only ∼9% of the F2 mutants showing the PVM mispositioning phenotypes after 32 d of L1 arrest, compared to the ∼70% penetrance of the phenotype among the mutants derived from homozygous mothers (Fig. 4a). We reason that either *dpy-19* mRNA is incredibly stable or the maternal *dpy-19* mRNA is only needed in the very early stage of L1 development before the starvation-induced arrest takes effect.

Since the 3′UTR often regulates the stability of mRNAs, including maternally deposited mRNAs (Mishima and Tomari 2016; Dai et al. 2019), we next tested whether *dpy-19* 3′UTR has similar effects. We swapped the 96-bp sequence encoding the *dpy-19* 3′UTR with a 73-bp fragment encoding the *mig-21* 3′UTR at the endogenous *dpy-19* locus through CRISPR/Cas9-mediated gene editing (Fig. 4b). The rationale was that the *mig-21* mutants caused similar PVM phenotypes but did not show any maternal effects. This swap did not affect *dpy-19* function, since we did not observe any phenotypes in the *dpy-19* mutants with *mig-21* 3′UTR. We then generated heterozygotes carrying 1 *dpy-19 (3′UTR swapped)* allele and 1 *dpy-19(−)* allele and found that among their *dpy-19(−)* homozygous mutant progeny, only 2.6% (*n* = 228) showed the PVM positioning defects, suggesting that the 3′UTR of the gene does not contribute significantly to the maternal rescue (Fig. 4c). Furthermore, the starvation-induced L1 arrest did not cause any stronger effects on the phenotype in the *dpy-19(−)* animals produced by the *dpy-19(−)/dpy-19(3′UTR swapped)* heterozygotes compared to the mutants derived from *dpy-19(−)/+* mothers (8% vs 9%), supporting the idea that the *dpy-19* 3′UTR does not play a significant role in stabilizing its mRNA.

Lastly, we hypothesized that certain RNA-binding proteins may be involved in stabilizing the maternal *dpy-19* mRNA and preventing it from degradation during embryogenesis. We chose to test 4 genes, including *cey-4* (homolog of mammalian YBX1 and YBX2), *gtbp-1* (homolog of G3BP1), *imph-1* (homolog of IGF2BP), and *pab-2* (homolog of PABPC1), because of their functions in stabilizing target mRNAs and interacting with m^6^A- or m^5^C-modified RNAs (Boo and Kim 2020; Nombela et al. 2021). Although whether these RNA modifications exist on the mRNAs of *C. elegans* is still under debate, recent studies found that m^6^A modification on the pre-mRNA of *sams-3* regulates mRNA splicing (Mendel et al. 2021; Watabe et al. 2021). Thus, although these modifications may not be widely present in mRNAs, they may still occur on selected mRNA species. We first crossed the null alleles of the above genes with *dpy-19(−)* and confirmed that the PVM mispositioning phenotype was not affected in the double mutants. We then conducted the test for maternal rescue in the genetic background that is depleted of these RNA-binding proteins (Fig. 4d). We found that only the depletion of *pab-2* suppressed the maternal rescue in ∼4% (*n* = 152) of F2 *dpy-19(−)* mutants arising from heterozygous mothers, while the loss of the other 3 had no effects (Fig. 4e). *pab-2* codes for a homolog of PABPC1, which not only binds to the 3′ poly(A) tail of mRNAs (Sachs and Davis 1989 ) but also interacts with m^6^A- and m^5^C-modified mRNAs through IGF2BP and YBX1, respectively (Yang et al. 2019; Zhang et al. 2021). The fact that the loss of *imph-1/IGF2BP* and *cey-4/YBX1* did not produce similar effects suggests that the 2 RNA modifications may not be involved in stabilizing the *dpy-19* mRNAs, or each alone is not sufficient. The weak effects of *pab-2* may also be due to the redundancy with *pab-1*, which is an essential gene.

Given that the various treatments and genetic perturbations above did not significantly disrupt the maternal rescue of *dpy-19(−)* mutants, we reason that the maternal *dpy-19* mRNA may be stabilized by multiple and potentially redundant mechanisms or some unknown factors.

## Discussion

By tracking the Q lineage descendants in *C. elegans*, we showed that neuroblast migration in postembryonic neurodevelopment is regulated by maternally deposited *dpy-19* mRNAs. These mRNAs are so stable that they can be passed down from the zygote all the way to the embryonically born Q neuroblasts through 10 rounds of cell divisions that take ∼720 min. These mRNAs then persist throughout the rest of embryonic development and the first few hours of larval development to regulate the initial polarization of the Q cells. Thus, the maternal *dpy-19* mRNAs can carry out their functions 15 to 16 h after the cleavage of the zygote, which suggests remarkable perdurance given that the life cycle of the animal is merely 3 d. Although we found that the 3′UTR of *dpy-19* and the poly(A)-binding protein PAB-2 contribute to *dpy-19* mRNA stability, their contributions were relatively minor. It remains unclear what mechanisms allow *dpy-19* mRNA to survive the maternal mRNA clearance and persist throughout development. One hypothesis is that some *cis*-regulatory elements in the coding region of *dpy-19* mRNA may recruit *trans*-acting RNA-binding proteins to help stabilize the mRNA. Although previous studies found that the motifs located in the coding region often promote mRNA decay (e.g. coding region determinants in *c-fos* and *c-myc* mRNAs) (Chen et al. 1992; Yeilding and Lee 1997), it is plausible that some motifs may promote stability. In particular, if the motif recruits RNA-binding proteins that undergo phase separation to form RNA granules, *dpy-19* mRNA inside the granule may be protected from degradation, since RNA granules are known to store translationally repressed mRNAs for a long period of time until the right condition occurs (Putnam et al. 2023). Finally, a zygotic copy of *dpy-19(+)* can also rescue the Q neuroblast migration defects in *dpy-19* mutants, suggesting that the maternal *dpy-19* mRNA is sufficient but not necessary for Q cell migration. This finding also suggests that the postembryonic neuronal development is a robust process redundantly regulated by both maternal and zygotic genes.

A few other examples of maternal genes regulating postembryonic development came from early screens for maternal-effect viable mutants in *C. elegans* (Hekimi et al. 1995). For instance, the *clk* genes regulate embryonic and postembryonic development, reproduction, and rhythmic behaviors, and their mutant phenotypes are fully rescued by maternal contributions (Benard et al. 2001; Burgess et al. 2003); *clk-1* codes for a 5-demethoxyubiquinone hydroxylase, and *clk-2* codes for a telomere maintenance protein. Another example is the gene *mau-2*, which codes for a protein with predicted sister chromatid cohesin loading and dsDNA-binding activities. *mau-2* mutants showed defects in larval development, locomotion, neuronal migration, and axonal guidance, which were all rescued by maternally deposited *mau-2* mRNAs (Takagi et al. 1997). MAU-2 regulates the positioning and axonal guidance of both embryonically and postembryonically derived neurons including the TRN subtype AVM, and the AVM guidance defects in *mau-2* mutants can also be rescued through a zygotic copy of the gene expressed cell-autonomously (Benard et al. 2004). Given that 80 of the 302 neurons in the *C. elegans* adult hermaphrodite are derived postembryonically, we suspect that the differentiation of many neurons in the *C. elegans* nervous system may be subjected to both maternal and zygotic controls.

*dpy-19* codes for a conserved C-mannosyltransferase that attaches an α-mannose to the tryptophan residue in the TSR domain of the substrate (Buettner et al. 2013). C-mannosylation regulates protein folding and secretion (Inai et al. 2020). In the Q cells, DPY-19 glycosylates the TSRs in UNC-5 and MIG-21 for their stability and secretion (Buettner et al. 2013); MIG-21 subsequently regulates Q cell polarization and migration. Interestingly, the DPY-19 function in neuronal migration appears to be conserved across species, as Watanabe et al. (2011) showed that the mouse homolog DPY19L1 is highly expressed in the developing glutamatergic neurons in the mouse embryonic cerebral cortex and regulates their radial migration. In humans, the deletion of DPY19L2 causes globozoospermia, a male infertility condition characterized by round-headed spermatozoa due to defective sperm head elongation and acrosome formation (Harbuz et al. 2011; Koscinski et al. 2011). Mechanistic studies in mice found that DPY-19L2 was located to the inner nuclear membrane of the spermatids and helped anchor acroplaxome to the nuclear envelope (Pierre et al. 2012). Whether mammalian *dpy-19* homologs also show unusual mRNA stability and have maternal effects awaits further investigations.

## Supplementary Material

jkaf151_Supplementary_Data

## Data Availability

Strains and plasmids are available upon request. The authors affirm that all data necessary for confirming the conclusions of the article are present within the article, figures, and tables. Supplemental material available at G3 online.
