# Supplementary material for: Maternal effects on postembryonic neuroblast migration in Caenorhabditis elegans
Source: G3 (Bethesda). 2025 Jun 30;15(9):jkaf151. doi: 10.1093/g3journal/jkaf151 (PMC12405876; doi:10.1093/g3journal/jkaf151)
Supplement: jkaf151_Supplementary_Data [file jkaf151_supplementary_data.zip › Figure_S1-S5_G3-2025-406005.docx]

**Supplemental Information**

**Table S1. Strains, constructs, and primers used in this study.**

**Table S2. Genes that code for proteins containing the “WXXWXXW” motif.**

**Figure S1. Mapping of *unk88*.**

**Figure S2. Maternal rescue of *dpy-19* mutants only lasted for one generation.**

**Figure S3. Maternal rescue of the AVM mispositioning in *dpy-19* mutants.**

**Figure S4. Maternal rescue of the Dumpy phenotype in *dpy-19* mutants.**

**Figure S5. Expression of DPY-19 proteins in mid- to late-stage embryos.**


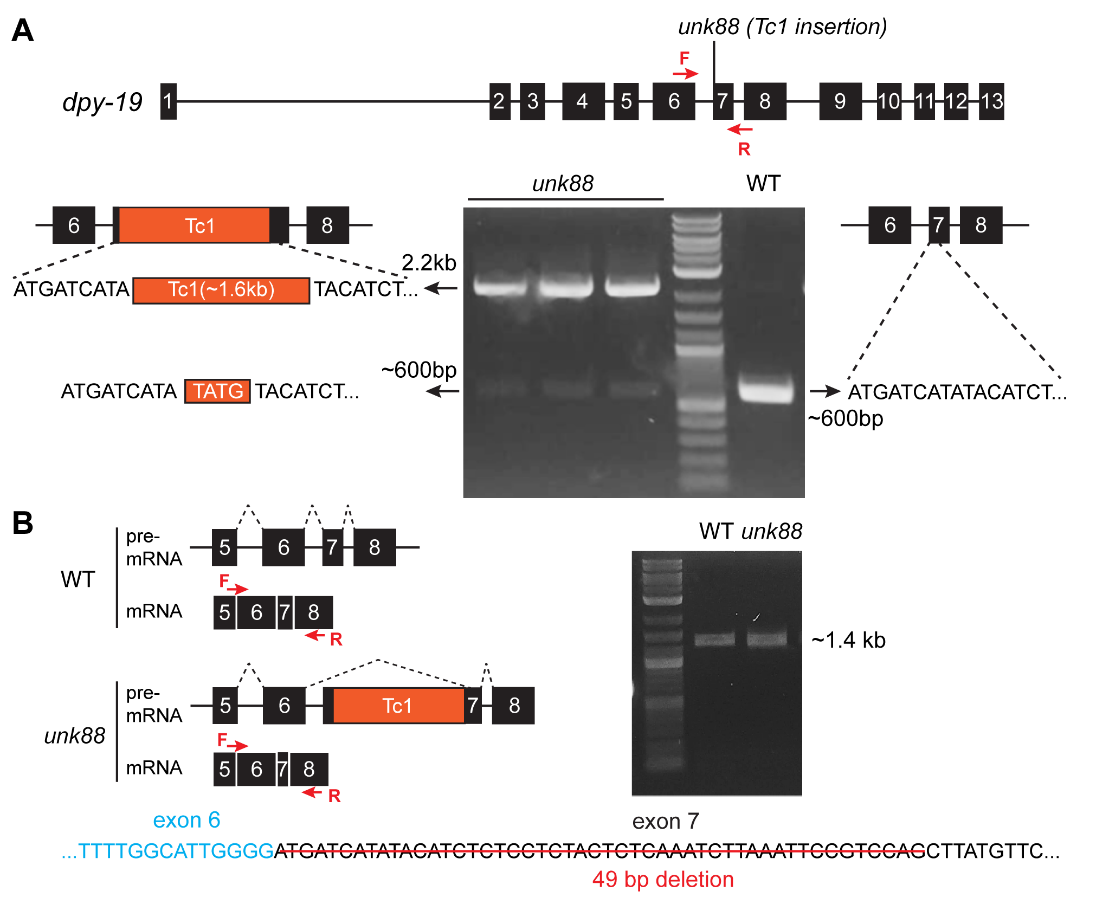


**Figure S1. Mapping of *unk88*.** (A) Tc1 transposon insertion in *dpy-19* exon 7 identified by whole-genome resequencing and Sanger sequencing in the *unk88* allele. Using the primers that bind to exon 6 and exon 8 (F and R in red), we amplified a ~600 bp band in the wild-type animals and a 2.2 kb band in *unk88* animals. However, there was a weak ~600 bp band that contains a 4-nt (TATG) insertion, which likely resulted from the self-excision of the Tc1 transposon, in the *unk88* animals. (B) RT-PCR results using a forward primer spanning the exon 5 and 6 junction and a reverse primer that binds to exon 8 (F and R in red). Unexpectedly, both wild-type and *unk88* animals gave a ~1.4 kb band. Sanger sequencing of the PCR product amplified from the cDNA of mixed stage animals led to the discovery of a 49-bp deletion in the cDNA of *unk88*, which caused a frameshift. We reasoned that the Tc1 was skipped during mRNA splicing due to cryptic splicing sites.


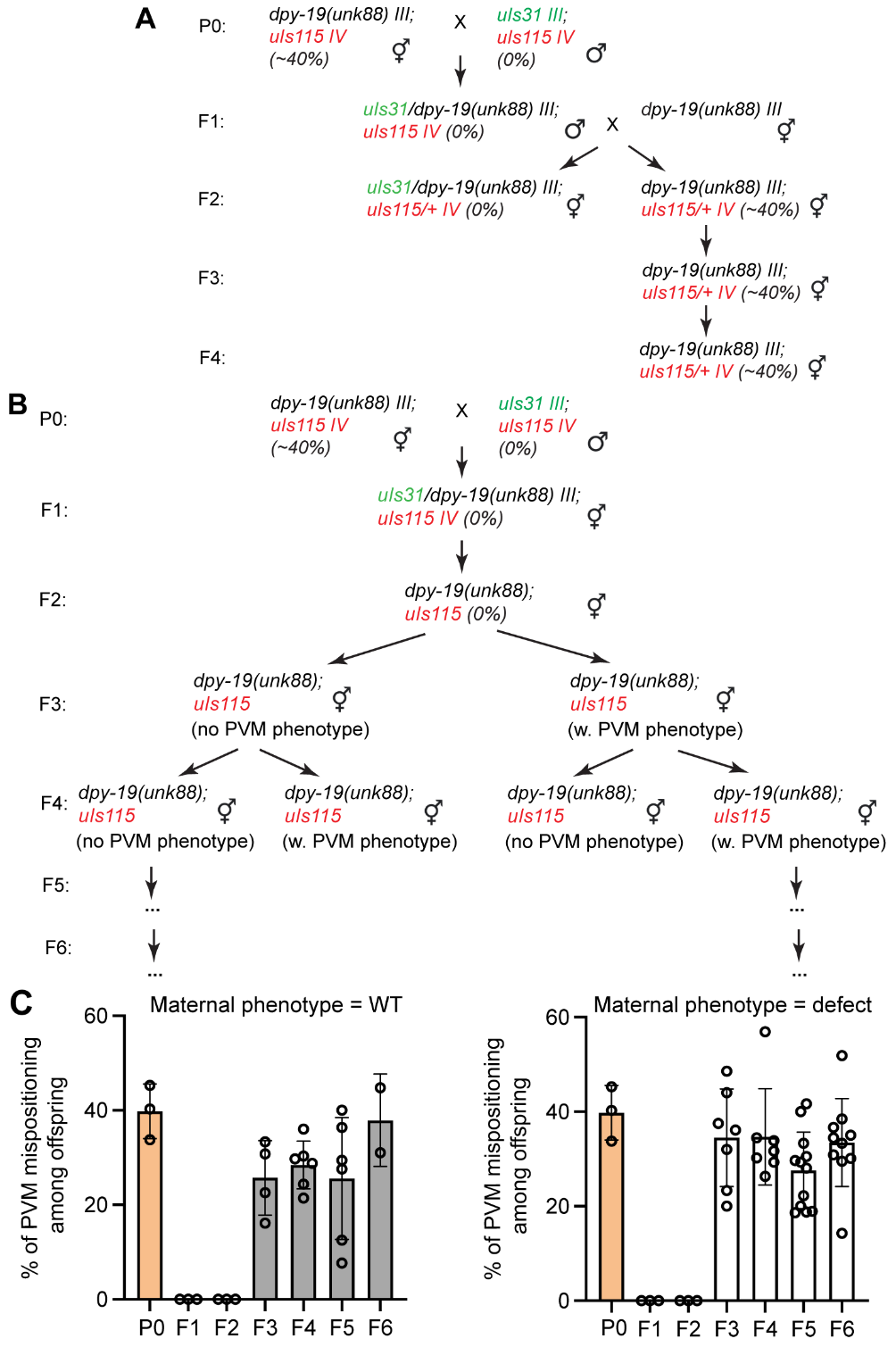


**Figure S2. Maternal rescue of *dpy-19* mutants only lasted for one generation.** (A) A cross scheme to examine the paternal rescue of *dpy-19(unk88)* mutants by crossing *dpy-19/uIs31* males with *dpy-19* hermaphrodites. The homozygous *dpy-19* progeny derived from heterozygous fathers showed PVM mispositioning phenotype, suggesting a lack of paternal rescue. (B-C) A cross scheme to examine the influence of maternal phenotype on the phenotype of the offspring. From the F3 generation onward, animals were separated into ones with and without PVM mispositioning phenotype. The percentage of animals showing the phenotype in each generation from F4 to F6 were recorded. Each data point represents a biological replicate. For (C), at least 50 animals were examined at each generation.


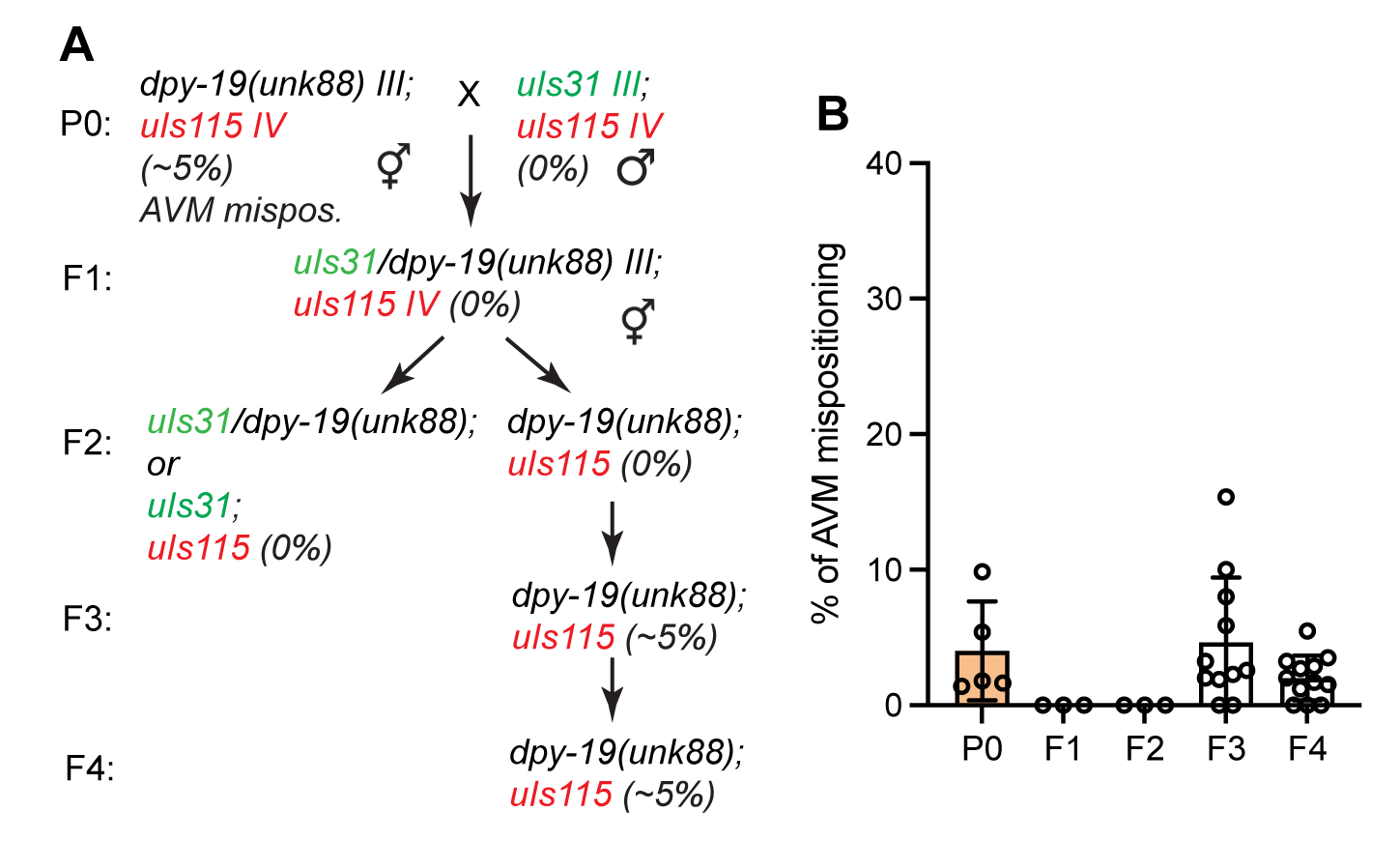


**Figure S3. Maternal rescue of the AVM mispositioning in *dpy-19* mutants.** (A) A cross scheme for the maternal rescue experiment. *uIs31[mec-17p::GFP]* is integrated on chrIII and used as a marker for chrIII not carrying the *dpy-19* mutation. (B) Percentage of animals showing AVM mispositioning in each generation according to the labeling in (A) for *dpy-19(unk88)* mutants*.* For each data point, N > 50.


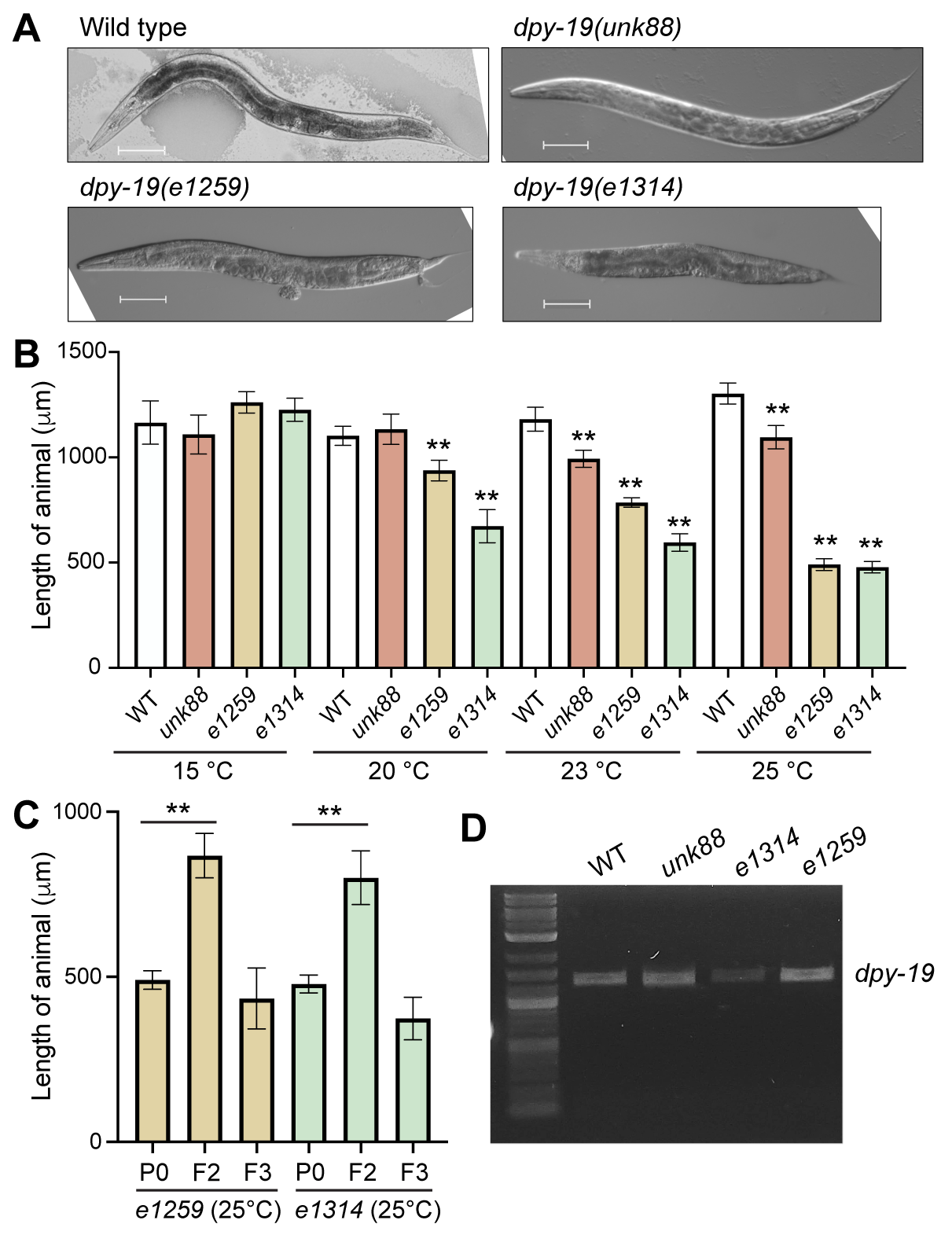


**Figure S4. Maternal rescue of the Dumpy phenotype in *dpy-19* mutants.** (A) The morphologies of wild-type and various *dpy-19* mutants grown at 20^o^C. Scale bar = 100 µm. (B) The length of the wild-type animals and various *dpy-19* mutants grown at the indicated temperatures. Double asterisks indicate significant difference in comparison with the wild-type animals at the same growing temperature in a post-ANOVA Tukey’s test. At least 10 animals were measured. (C) The length of *dpy-19(e1259)* and *dpy-19(e1314)* animals from a cross scheme similar to the one in Figure 1E. P0 and F3 represent mutants that are M-, while F2 are mutants that are M+. At least 15 animals were measured. (D) RT-PCR for *dpy-19* from cDNA libraries constructed using mixed stage animals from various strains.


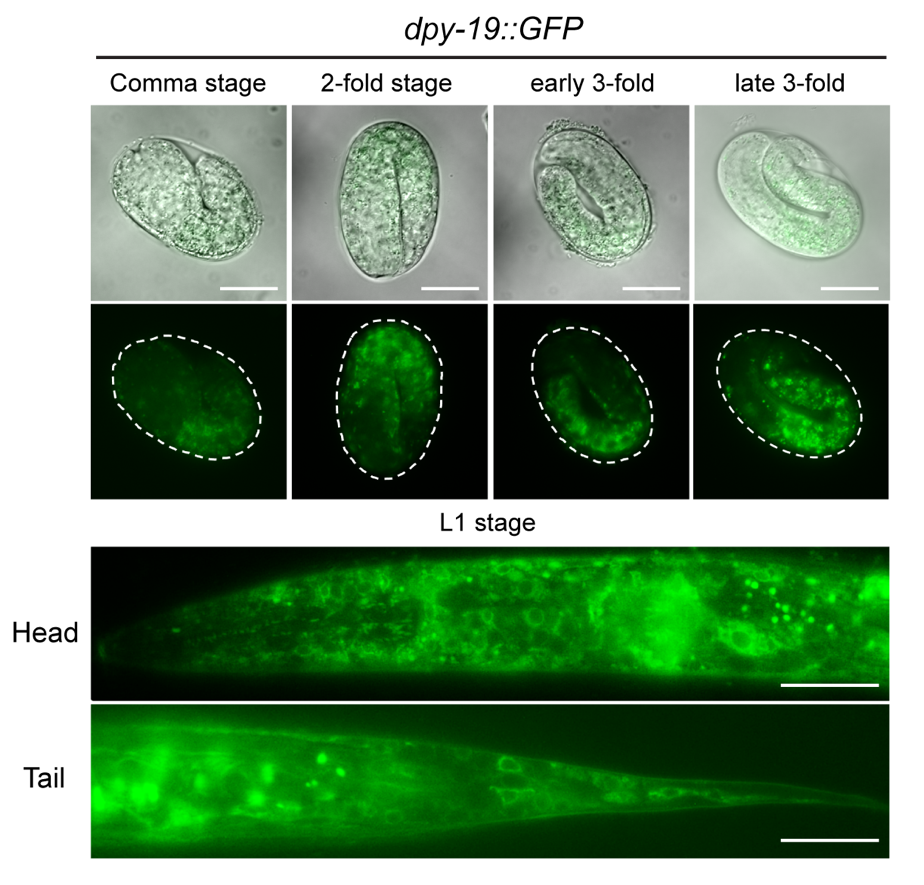


**Figure S5. Expression of DPY-19 proteins in mid- to late-stage embryos.** DPY-19::GFP expressed from an endogenous GFP knock-in allele *hu257[dpy-19::gfp::SEC::3xflag]* started showing GFP signal from comma stage embryos, and the signal persisted into late-stage embryos and early larvae. Scale bar = 20 μm.
